# Peroneal muscle activity during different types of walking

**DOI:** 10.1186/s13047-018-0291-0

**Published:** 2018-09-03

**Authors:** Rok Bavdek, Anže Zdolšek, Vojko Strojnik, Aleš Dolenec

**Affiliations:** University of Ljubljana, Faculty of Sport, Gortanova 22, 1000 Ljubljana, Slovenia

**Keywords:** Gait, EMG, Ankle joint, Peroneal muscles, Foot

## Abstract

**Background:**

As the most common form of movement, walking happens not only on flat but also on uneven surfaces, where constant loss and regaining of balance occur. The main balancing function of the ankle joint is performed by tibial muscles. When changing inclination in a frontal plane, an essential balancing function is performed by the peroneal muscles. One of the methods for improving the activity of peroneal muscles is walking with different foot placement. The objective of this study was to analyze the activity of the peroneal muscles when performing different types of walking.

**Methods:**

Sixteen healthy participants took part in this study, walking on a flat surface (NORM), on a medial incline ramp with the plantar surface of the foot fully placed on the surface (FULL), and on a medial incline ramp with elevated lateral part of the foot (LAT). We monitored the changes of EMG signals in peroneus longus (PL), peroneus brevis (PB), tibialis anterior (TA), soleus (SOL), gastrocnemius medialis (GM) and gastrocnemius lateralis (GL) muscles. We monitored kinematic parameters (gait speed, stride length, contact time, foot position). The parametric ANOVA test and a non-parametric Friedman test were used at an alpha level of 0.05.

**Results:**

This study shows that the EMG activities of peroneal muscles increases when walking on the medial incline ramp. Statistically significant EMG differences were observed in the peroneal muscles, TA and GL muscles. We observe a very high percentage of normalized EMG value of the PL muscle in LAT walking. Walking on a medial incline ramp impacts the foot position, contact time, and stride length but not the gait speed.

**Conclusions:**

Walking on a medial incline ramp could be an effective exercise to improve the neuro-muscular function of the peroneal muscles and, therefore, might be a suitable exercise for people with weakened ankle evertors.

## Background

Due to the change of the ankle position in its frontal plane, locomotion over uneven surfaces (inclines, snow, sand, grass, gravel, etc.) leads to changes in the activation of the tibial muscles and, especially, the peroneal muscles [1–4], which primarily function as ankle evertors [5]. The main function of the peroneal muscles in walking is mediolateral stability [6, 7] and preventing involuntary ankle inversion at foot strike [8, 9]. The activation of peroneal muscles in walking is the greatest during single limb support in the mid-stance phase when the subtalar joint is in a pronated position; the peroneal muscles and also active in the push-off phase of the stride [7, 10]. Peroneus longus (PL) and peroneus brevis (PB) muscles, which are the subject of this research, have different inserts: the PB inserts on the fifth metatarsal while the PL inserts on the first metatarsal bone [5]. The function of the PL tendon includes stabilizing and supporting the longitudinal arch by pulling the base of the first metatarsal inferiorly and aiding in the plantarflexion of the foot [11–14]. Studies also assume that low PL activity is associated with flat-arched feet as PL generates a first-ray plantarflexion moment and a forefoot plantarflexion moment during its contractile activity. [15, 16]. In human movement, the peroneal muscles are essential at the moment of the individual’s sudden change of ankle position in the direction of inversion, which is also the most common reason for lateral ankle sprain which may cause a clinical syndrome functional ankle instability (FAI) [17, 18]. As evertor muscle strength is associated with FAI [19], one of the methods for the improvement of the neuro-muscular function of peroneal muscles is strength training exercise. Evertor orientated strength training improves evertor muscle strength, joint position sense and also reduce reaction time during walking [20–22]. One way to increase the activation of peroneal muscles so as to prevent sudden imbalance in the direction of inversion is walking transverse on an incline, which increases the activity of peroneal muscles and tibialis anterior (TA) muscle [4, 23]. To avoid the possibility of further injury in the direction of the inversion during walking, the peroneal muscles need resistance through the increase of torque in the direction of ankle eversion. This can be achieved by adding a weight to the dorsal-lateral side of the foot in an attempt to increase co-activation of peroneal muscles and TA, and consequently to stabilize the ankle [24]. However, when searching for suitable exercises for strengthening ankle evertors that are simple to use, accessible and without the use of any load, walking on a medial incline ramp with an elevated lateral part of the foot may be a promising method to increase the electromyographic (EMG) activity of the peroneal muscles.

**Fig. 1:**
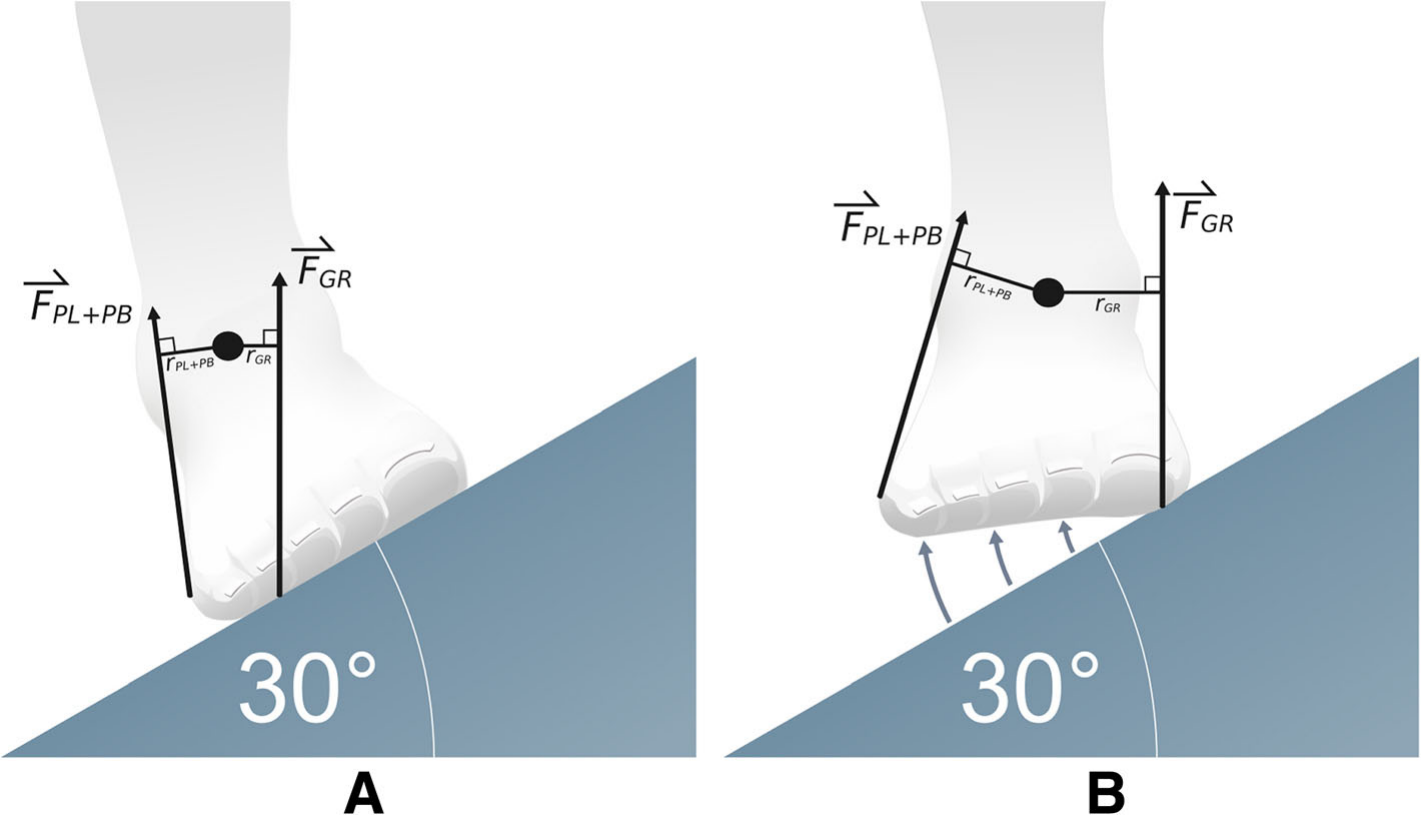
Demonstration of both walks on a medial incline ramp; **a**. Walking on an incline ramp, plantar surface of the foot is fully placed on the walking surface – FULL; **b**. Walking on an incline ramp, lateral part of the foot elevated – LAT. Force, peroneal muscles - ${\overset{\rightharpoonup}{F}}_{PL+ PB}$; Force, ground reaction - ${\overset{\rightharpoonup}{F}}_{GR}$; Lever arm, peroneal muscles - *r*_*PL* + *PB*_; Lever arm, ground reaction force - *r*_*GR*_

The biomechanical model shows the foot position when it is fully placed on the inclined walking surface (Fig. 1a). The ground reaction force (GRF) is distributed on the lateral side of the inverted foot [25], therefore only a small peroneal muscle force is needed to keep the foot in equlibruium. When the lateral part of the foot is elevated, the foot effectively touches the inclined ramp only with the medial part (Fig. 1b), thus causing considerably greater torque on the foot due to greater GRF lever arm. In order to keep the foot in equilibrium with the elevated lateral part, the GRF torque must be balanced with the torque of equal magnitude in the opposite direction. It originates from the lateral side and is caused by the peroneal muscles.

The objective of this study was to determine the differences in the EMG activity of the peroneal muscles (PB and PL) in normal walking (NORM), walking on a medial incline ramp (30° incline) when the full plantar surface of the foot is placed on the surface (FULL) and walking with the elevated lateral part of the foot (LAT). Based on previous research indicating that walking on uneven terrain increases the ankle stiffness of tibial muscles’ EMG, we monitored four other tibial muscles. We hypothesized that (a) walking on the medially inclined ramp with elevated lateral part of the foot additionally increases the activity of the peroneal muscles over full foot contact incline walking; (b) walking on the medially inclined ramp increases the activation of the PL, PB, TA, GL, GM, and SOL.

## Methods

### Participants

This study includes 16 participants (8 men age 26.3 ± 3.7 yrs.; body height 1.81 ± 0.05 m; body mass 82.4 ± 11.0 kg, and 8 women age 29.0 ± 6.7 years old; body height 1.76 ± 0.69 m; body mass 61.6 ± 16.5 kg). In this measurement process, all the participants were injury free. Before the measurements were performed, the protocol was explained to them, as well as their rights regarding their participation in the study, which were in accordance with the Helsinki Declaration.

### Protocol

First, measuring equipment was placed on the participants: surface electrodes, IMU, accelerometer, and a miniature laptop as a data logger. After the equipment placement, the measurements of maximal voluntary isometric contractions (MVIC) were performed (plantar flexion, dorsal flexion, ankle eversion and inversion), which were used for normalizing EMG signal. All MVIC were performed against resistance. Participants were verbally encouraged to give maximal effort and hold the contraction for approximately five seconds. With the first isometric device, ankle eversions were measured for normalization of m. peroneus longus (PL) and m. peroneus brevis (PB). The subjects were sitting on chairs with adjustable height so that the angle of their knees and hips was at a 90°. The knees were fixed to exclude any hip movement. With the second isometric device, the subjects performed plantar and dorsal flexion of the foot for normalization of m. soleus (SOL) and *M. tibialis* anterior (TA). The subjects were sitting with their knees fixed, and the angle of their hips and knees was 90°. To determine MVIC for m. gastrocnemius medialis (GM) and lateralis (GL), a stationary barbell was used for the participant to push up during standing isometric plantarflexion, as according to Hebert-Losier [26]. The measurements were performed in random order. Every MVIC measurement was performed twice with a two-minute rest period between test.

### Walking measurements

**Fig. 2:**
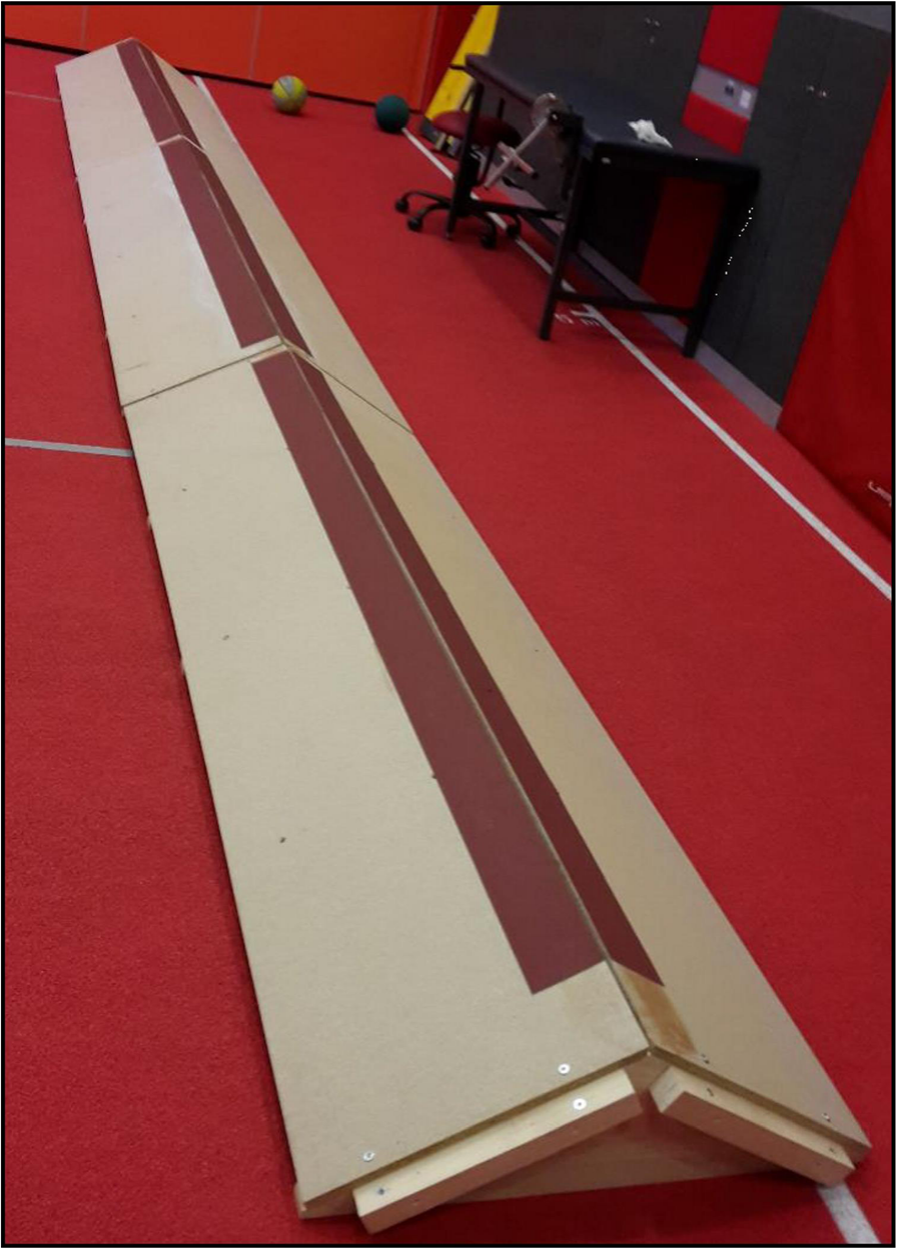
Medial incline ramp

Participants performed three different types of walking at their self-selected walking speed: walking on a flat surface (NORM), walking on a medial incline ramp with a 30° inversion (FULL) (Fig. 1a), and walking on a medial incline ramp with a 30° inversion with additional instruction, that the participants maintain the lateral part of the foot elevated, supporting themselves only with the medial part of the foot (LAT) (Fig. 1b). These three walking types were always performed with both legs (left and right), but only the right leg was recorded. The participants underwent measurements of walking in random order. A single walking task was 33-m long and consisted of four lengths on the incline ramp. The incline ramp was 8.25 m long, made of wood, and lined with sandpaper, which prevented the foot from sliding off (Fig. 2). A 30-s rest period was provided between each task repetition.

### Kinematic recordings

During walking, a 3-D sensor for measuring acceleration is taped to the heel of the participant (Biovision, Wehrhein, Germany), to measure the contact time. To control the foot position and the measurements of the lengths of strides, Panasonic DMC-FZ200 camera (300 fps; Panasonic UK, Great Britain), was used. It was placed in a sagittal position to the participant. To measure the gait speed, expressed in m/s^− 1^, Stalker’s type hyper-frequency radar (Stalker Professional Radar, Radar Sales, Plymouth, MA, USA) was used. It was placed in a frontal position in relation to the direction of walking. The participant was tracked with a camera and radar from the start to the end of the measuring interval.

The foot position during walking was controlled and monitored with inertial measurement unit (IMU) (MTx, Xsens Technologies B.V., Enschede, Netherlands), which was placed on the dorsal side of the right forefeet. Foot position was observed in the x-axis (*Roll)* and was expressed in degrees (*°*).

### Surface electromyographic recordings

First, points for the EMG electrodes placement were determined according to the SENIAM [27]. Bipolar Ag/AgCl surface electrodes (Kendall, Neustadt/Donau, Germany), 10 mm in diameter and with a 20 mm inter-electrode distance were placed on the belly of next muscles: peroneus longus, peroneus brevis, tibialis anterior, soleus, gastrocnemius medialis, and gastrocnemius lateralis of the right leg. To minimize any electrode movement, they were fixed using adhesive tape. A sixteen-channel EMG system with a differential amplifier (Biovision, Wehrhein, Germany), was used to record muscle activation. The EMG and acceleration data were transmitted to a UMPC miniature notebook computer (Viliv, Yukyung Technologies Corp., South Korea) where the analogue data were sampled at 2000 Hz (16-bit resolution) and stored for analysis.

### Data reduction

For processing the EMG signal, Labchart 7 was used (AD Instruments, Dunedin, New Zealand). In processing the data of MVIC, the signal was digitally filtered (20 Hz/500 Hz, bi-directionally) to remove a base line shift. Later, a full-wave rectified signals were taken and smoothed with a median filter (Window width 501 samples). In each of the six muscles, the maximal EMG signal value was taken and used for the normalization of the walking. The same procedure of processing the EMG signal in walking was used (filtering, full-wave rectified and smoothing).

The average of 15 correctly performed measurements of strides made with the right foot was analysed. The average EMG amplitudes during the stance phase (from the moment of foot strike to the moment the foot left the surface) were used for statistical tests. Contact time was also measured (expressed in seconds). Only strides with full heel-toe technique were used for further analysis. The contact time for each step was read from the acceleration signal of the right leg. Heel strike was detected as the time of occurrence of the nearest main peak of the vertical heel acceleration. Toe-off was recognized as when the acceleration happened after raising the heel from the floor. The accuracy of contact time achieved from the acceleration signal was also monitored by counting photos from the high-speed cameras during the foot’s contact with the ground. An accuracy control was made for the two analysed steps in each condition. The steps for control were chosen randomly from analysed steps. The contact times of all analysed strides in three types of walking were averaged, and the standard deviation was calculated for each participant. The stride length, expressed in meters, and considered to be two consecutive steps, from heel to heel (2-D kinematics), was analysed with Kinovea 0.8.15 software (*Joan Charmant & Contributors, Bordeaux, France).*

### Statistical analysis

For all parameters (EMG, kinematic parameters), average values were calculated, and the Shapiro-Wilk test was used to check the normal distribution. At normally distributed variables (*p* > 0.05), the parametric ANOVA test for repetitive measurements was used. Post-hoc tests, using a Bonferroni correction, were used to determine pairwise differences when the ANOVA showed an overall significant F statistic. If the data were not normally distributed, a non-parametric Friedman test was used. To compare two non-parametric parameters, a Wilcoxon signed ranks test was used when necessary. Statistical significance was accepted at an alpha level of 0.05, except in the case of multiple Wilcoxon test, for which the alpha level was adjusted to 0.017 to avoid inflation of Type I error.

## Results

### Kinematic parameters in walking

Kinematic parameters in all three walking types are presented in Table 1. There were no statistically significant differences among the conditions in walking speed, F (2,10) = 4.1, *p* > 0.05. Statistically significant differences were obtained in stride length, contact time and foot roll (F (2,14) = 46.7, *p* < 0.001; F (2,14) = 12.1, *p* < 0.01; χ^2^ (2) = 20.7, *p* < 0.001; respectively). The stride lengths were the longest in NORM (mean ± SD: 1.306 ± 0.027 m), shorter in FULL (1.135 ± 0.033 m), and the shortest in LAT (0.930 ± 0.040). Contact times were the longest in FULL (0.74 ± 0.07 s) and statistically significantly different from NORM (0.74 ± 0.07 s, *p* < 0.05). No statistically significant difference was observed between FULL and LAT. The smallest roll occurred in NORM (1.65 ± 1.06 °) and the greatest in FULL (24.25 ± 8.03°, *p* < 0.001). By elevating the lateral part of the foot in LAT, the angle of the foot in the x-axis reached 6.33 ± 7.57° and was statistically significantly different from NORM (*p* < 0.01) and FULL (*p* < 0.01).

**Table 1:** Kinematic parameters of three types of walking

	NORMAL	FULL	LAT	ANOVA or Friedman test	Post-hoc comparisons
Gait speed (m·s)^− 1^	0.89 ± 0.12	0.83 ± 0.1	0.76 ± 0.09	F = 4.1	/
Stride length (m)	1.306 ± 0.027	1.135 ± 0.033	0.930 ± 0.040	F = 46.7 ***	NORM > FULL *** NORM > LAT *** FULL > LAT ***
Contact time (s)	0.66 ± 0.09	0.74 ± 0.07	0.69 ± 0.13	F = 12.1*	NORM < FULL *
Foot position - Roll (°)	1.65 ± 1.06	24.25 ± 8.03	6.33 ± 7.57	χ^2^ = 20.7 ***	NORM < FULL *** NORM < LAT ** FULL > LAT **

* - *p* ≤ 0.05; ** - *p* ≤ 0.01; *** - *p* ≤ 0.001

### EMG parameters

**Fig. 3:**
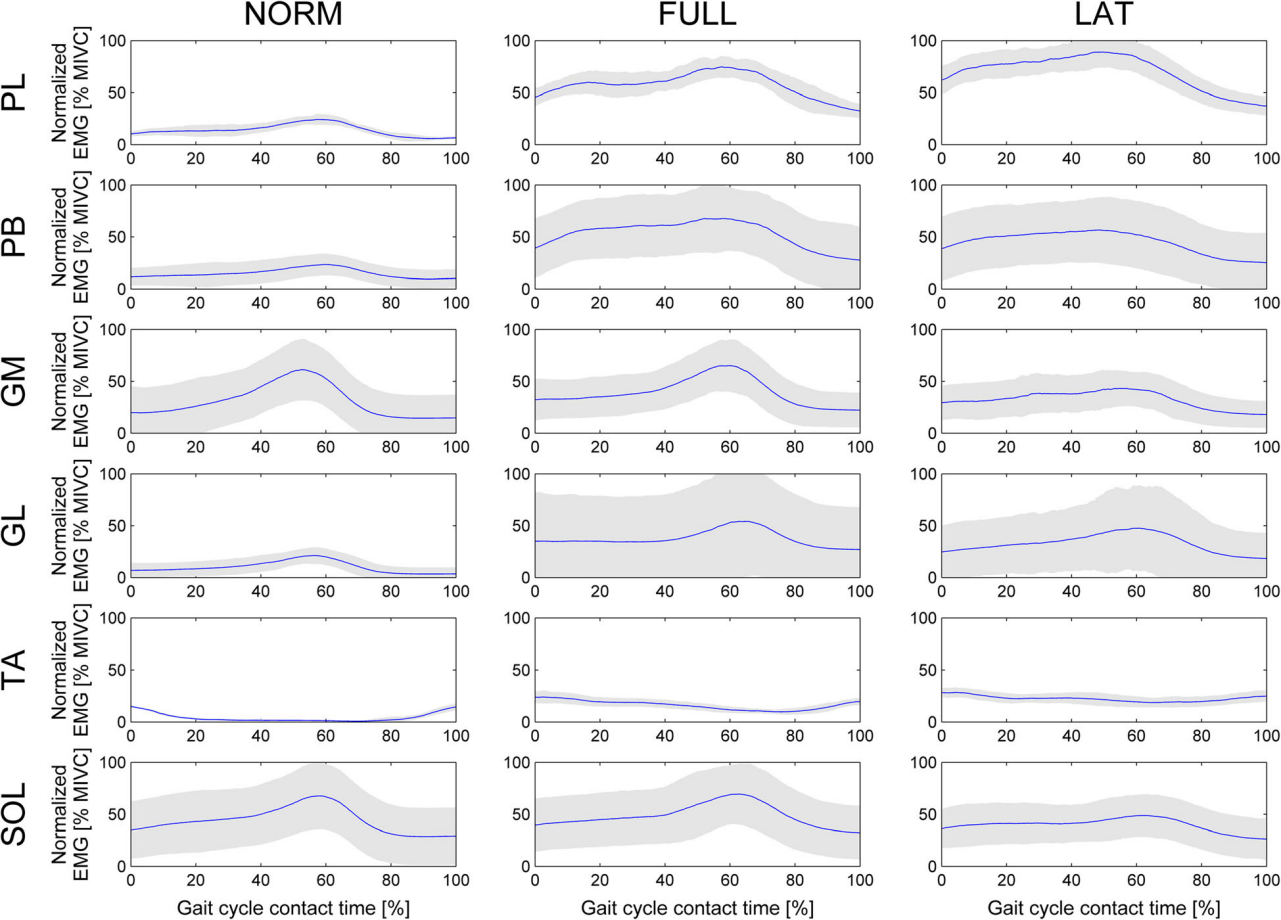
Ensemble averaged EMG curves of the analysed muscles during contact phase in three walking types. NORM, FULL and LAT – see explanation in text. * *p* < 0.05; ** *p* < 0.01; *** *p* < 0.001

Figure 3 presents the ensemble averaged EMG curves of the EMG signals during the stance phase for the analysed muscles and for all three walking types. Visual inspection showed that variability of the EMG signals was the smallest in the TA and PL muscles (as later confirmed with mean EMG amplitudes and standard deviations) and that the curves followed similar shapes among walking types.

**Fig. 4:**
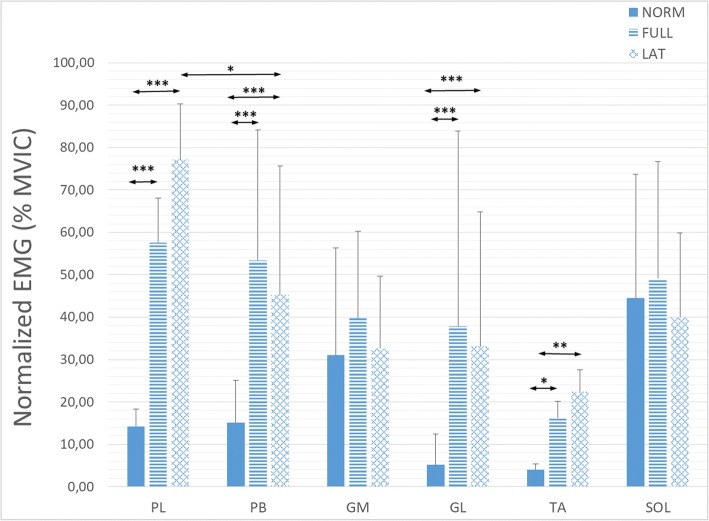
Mean normalized EMG amplitudes of analysed muscles during stance phase in three walking types; NORM, FULL and LAT – see explanation in text. * *p* < 0.05; ** *p* < 0.01; *** *p* < 0.001

Statistically significant differences in the mean EMG amplitudes of the single muscles during stance phases among different walking types were obtained for PL, PB, GL, and TA (χ^2^ (2) = 27.1, *p* < 0.001; F (2,14) = 14.9, *p* < 0.001; χ^2^ (2) = 21.4, *p* < 0.001; χ^2^ (2) = 12.9, *p* < 0.01; respectively) but not for GM and SOL muscles (Fig. 4). Mean PL EMG amplitudes in LAT and FULL conditions were statistically significantly higher than in NORM (77.17 ± 13.20%, 57.59 ± 10.57% and 14.22 ± 4.05%, *p* < 0.001, *p* < 0.01, respectively). However, there are no statistically significant differences between FULL and LAT. Similar behaviour of mean EMG amplitudes were found in the PB muscle (NORM 15.11 ± 10.02%, FULL 53.40 ± 30.72%, LAT 45.26 ± 30.28%). However, the mean EMG amplitude during LAT was statistically significantly greater in PL than in PB (*p* < 0.05). Mean GL EMG amplitudes followed the PB trend, although with a smaller signal levels (NORM 5.15 ± 7.32%, FULL 37.86 ± 46.03%, LAT 33.13 ± 31.75%) and did not differ statistically significantly between FULL and LAT. Again, behaviour of mean the TA EMG amplitudes showed the same trend as in PL, but at the lowest activation level of all analysed muscles (NORM 4.05 ± 1.29%, FULL 16.17 ± 3.91%, LAT 22.45 ± 5.14%).

## Discussion

The main objective of the study was to determine how different types of walking affect the activation of peroneal muscles. The results showed significantly increased activation of both peroneal muscles, as well as anterior tibial and lateral gastrocnemius muscles when walking on a medially inclined ramp. Although there were distinctive differences in foot kinematics between both inclined surface walking types, they were not significantly reflected in the mean EMG amplitudes during the stance phase of the most exposed muscles. Kinematic results show that the subjects were able to follow the instructions regarding different walking strategies at approximately the same gait speed in all three types of walking.

During FULL walking, increased activation of tibial muscles was observed. Muscle activation of PL increases with the increased medial incline of the walking surface. Lee et al. [23] found the greatest activity of PL at a 25° incline during standing and walking on the mediolateral ramp as the incline was gradually increased. Both peroneal muscles attempted to maintain equilibrium due to the incline and attempted to prevent excessive ankle inversion and offer resistance to the ankle inversion during the stance phase of gait. This is also in agreement with other studies [7, 10], as PL is responsible for establishing and maintaining balance during walking [7]. This supports our hypothesis that incline ramp walking increases peroneal muscle activity. However, when the subjects were walking with the elevated lateral part of the foot on the medial inclined surface, peroneal muscles were not additionally activated systematically. This was contrary to our expectations that the subjects would need to increase eversion torque to lift the lateral part of the foot. The inclination of the surface is a significant factor for walking strategy since injuries may take place if the inversion angle exceeds 35° [28]. In the present study, the inclination of 30° was used, which is still considered safe, but is close to a discomfort zone, resulting in cautious and stable foot placement [29]. From the kinematic point of view, the cautious foot placement may be seen through prolonged contact time in FULL walking and shortened stride length in comparison to NORM, as observed on unstable surfaces [3, 30]. Foot position results shows that the foot was the most inverted in FULL walking when participants put the entire plantar surface of their foot on the walking surface, which prolonged the contact time parameter. This is in contrast to LAT, in which the participants walked only on the medial part of the foot and tried to maintain the lateral part of the foot in a neutral position, which did not affect the contact time parameter. The conclusion regarding the kinematic parameters is that NORM, as the most stable form of human locomotion, had the longest stride length but the shortest, together with LAT, contact time.

When observing videos and results of foot position, the authors observe that in FULL walking participants placed most of plantar surface of the inverted (right) foot in the time of terminal stance phase of the opposite (left). The midstance phase and placing the whole body mass on the inverted foot was, therefore, performed when most of the plantar surface of the foot was already in contact with the incline surface. That resulted in relatively short eccentric contraction. FULL walking on an incline ramp forced participants to fully stretch their peroneal muscles, and also presented a new and challenging motor task for the participants. For that reason, it might be expected that the peroneal muscles’ activity would eventually reduce in a time due to motor learning and adjust (decrease) peroneal muscle activation if FULL walking would be considered as a standard exercise method. In LAT walking on an incline ramp, where constant eversion torque is presented, participants were instructed to hold the lateral part of their foot horizontally during walking. That included the single leg midstance phase, which might be recognized as a quasi-isometric contraction.

Fear and caution may additionally increase PL EMG amplitude through muscle coactivation [31]. We observed similarly increased mean EMG amplitudes of PL and TA muscles among walking conditions, which act as an agonist-antagonist pair to provide lateral foot stability [7, 32]. Greater TA activation was also seen in artificially performed foot eversion [24]. As the main effect was observed from NORM to FULL condition, this is an additional argument to assume that the inclination angle posed some discomfort to the subjects.

Another factor to affect tibialis muscles’ activation during FULL is stretch reflex. Nieuwenhuijzen and Duysens [29] found that (of the six lower leg muscles they analysed) the peroneal muscles showed the largest increases in amplitudes in a short-latency response (SLR) and a late-latency response (LLR) during anticipated inclined walking. Such behaviour suggests changed anticipatory strategies. However, as the subjects accommodated to the stimulus, the LLR responses decreased (habituation) but not the SLR.

**Fig. 5:**
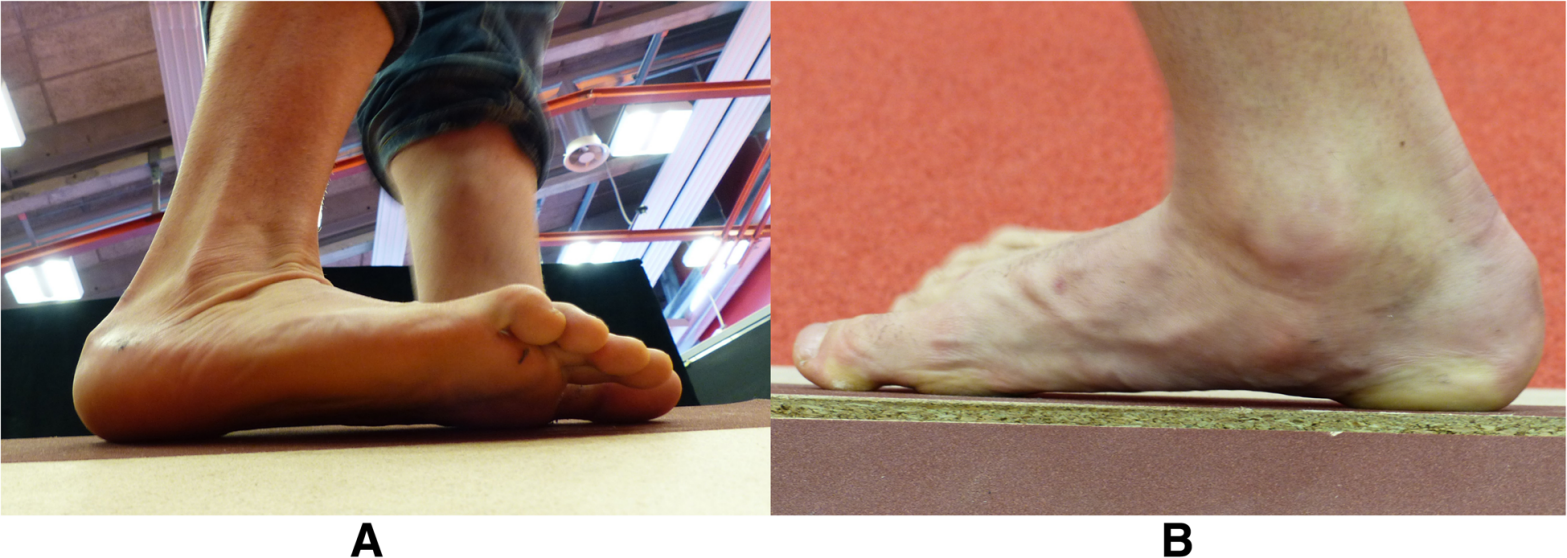
Foot position during walking on an incline ramp, lateral part of the foot elevated – LAT; **a**. Lateral view; **b**. Medial view

Comparing the EMG value of different muscles could be problematic as there is no golden standard for normalization methods [33]. With this in mind, speculations in further explanations were made. Although walking with an elevated lateral part of the foot did not additionally increased the activation of peroneal muscles systematically, PL was more activated than PB. It could be hypothesized that the reason was in different inserts of PL and PB [34] since their lever arm lengths are approximately equal [35, 36]. Nevertheless, the PL tendon, in contrast to PB, influences the shape of the foot arches [11–14, 16] as it inserts on the first metatarsal bone [5]. The medial longitudinal arch seems to be maintained during walking with elevated lateral part of the foot (Fig. 5) and consequently reflected a need for greater PL activation than PB. It seems that the subjects developed a walking strategy during the FULL condition to provide sufficient balance and security.

The triceps surae muscle is not meant primarily for lateral foot stability; however, it still may contribute to it. Some studies [3] showed a greater GM activation in walking on an uneven surface in comparison to regular walking. Others were more specific by noting that medial gastrocnemius is capable of inducing inversion torque [37]. As no inversion torque was induced in the present study, GM activation remained stable among the walking conditions. The role of GL in terms of inversion/eversion control may not be so clear. Some studies related GL with inversion torque [38]. In the present study, its activation was clearly increased in both inclined walking types assuming its averter’s role as assumed in a hypothesis. When the activation level is considered, SOL was the most active muscle from the plantar flexors during all three types of walking and was invariant to differences among them.

Can walking over a medially inclined surface serve as an exercise to strengthen the peroneal muscles? The study demonstrated very high mean (77% MVIC) EMG amplitude of PL in LAT walking. Its peak value was obtained during the mid-stance phase and was close to 100% of MVIC. This makes this type of walking interesting as an exercise to strengthen PL. As activation of lower leg muscles during FULL and LAT behaved in a similar way, FULL walking could serve the same purpose. However, there also were important differences between FULL and LAT. Walking on sufficiently inclined surfaces as during FULL may induce fear and caution [29] that increase muscle activation and coactivation to stabilize the ankle joint [31]. Increased stretch reflex was also observed to contribute to increased peroneal muscle activation during walking on a medially inclined ramp. Both are subject to habituation, which may decrease muscle activation over time, making such exercise less efficient for strengthening the muscles. This is less likely during LAT walking, as the muscles have to act against stable inversion torque. During mid-stance position in LAT walking, when the whole body weight was put on the foot, the EMG signal of PL almost reached 80% of MVIC. This suggests that slow walking or prolonged mid-stance phases may be even more effective for strengthening of the peroneal muscles. With putting on additional weight, the inversion torque can be further increased. To conclude, LAT walking seems to be a more efficient exercise for strengthening the peroneal muscles (especially PL) than FULL walking.

## Limitations

This study reports increases in EMG activity of the peroneal and other tibial muscles when healthy participants performed different types of walking. However, it would be interesting to observe if incline walk is applicable for patients with FAI or post-stroke patients whose health status affect activation of the peroneal and other tibial muscles while walking [18, 39]. Furthermore, patients with FAI also perform different kinematic patterns of walking [40]. Additional research is required to determine if medial incline ramp walking is appropriate for the above-mentioned patients. Finally, this study does not involve the EMG activity of hip or knee muscles, which are important while walking on medially inclined ramps [4, 41].

## Conclusions

In summary, walking on an incline ramp or changing incline in the frontal plane affects the position of the foot and impacts the activation of the peroneal muscles. This study proves an increased EMG activation of the peroneal muscles (PL and PB) when walking on an incline ramp. Incline walking impacts foot position, contact time, and stride length but not gait speed. During the LAT type of walking, the EMG activation of the PL muscle is higher than in the PB muscle. The findings of this study indicate that walking on the incline ramp affects the peroneal muscles and may potentially serve as a strengthening exercise for peroneal muscles.

## Abbreviations

°: Degrees
ANOVA: Analysis of variance
EMG: Electromyography
FAI: Functional ankle instability
FULL: Walking on a medial incline ramp with 30° inversion when the plantar surface of the foot is fully placed on the walking surface
GL: Gastrocnemius lateralis
GM: Gastrocnemius medialis
Hz: *Hertz*
IMU: Inertial measurement unit
kg: Kilogram
LAT: Walking on a medial ramp with 30° inversion with an elevated lateral part of the foot
LLR: Late-latency response
m: Meter
MA: Massachusetts
mV: Millivolt
MVIC: Maximal voluntary isometric contractions
NORM: Walking on a flat surface
PB: Peroneus brevis
PL: Peroneus longus
s: Second
SENIAM: Surface EMG for a non-invasive assessment of muscles
SLR: Short-latency response
SOL: Soleus
UK: United Kingdom
UMPC: Ultra-mobile personal computer
USA: United States of America
yrs.: Years
